# Organoid models of the tumor microenvironment and their applications

**DOI:** 10.1111/jcmm.16578

**Published:** 2021-05-25

**Authors:** Tao Xia, Wen‐Lin Du, Xiao‐Yi Chen, You‐Ni Zhang

**Affiliations:** ^1^ Department of Gastrointestinal‐Pancreatic Surgery Zhejiang Provincial People’s Hospital People’s Hospital of Hangzhou Medical College Hangzhou China; ^2^ Key Laboratory of Gastroenterology of Zhejiang Province Zhejiang Provincial People’s Hospital People’s Hospital of Hangzhou Medical College Hangzhou China; ^3^ Clinical Research Institute Zhejiang Provincial People’s Hospital People’s Hospital of Hangzhou Medical College Hangzhou China; ^4^ Department of Laboratory Medicine Tiantai People's Hospital Taizhou China

**Keywords:** cancer, drug screening, immunotherapy, organoid, personalized medicine, tumour microenvironment

## Abstract

A small percentage of data obtained from animal/2D culture models can be translated to humans. Therefore, there is a need to using native tumour microenvironment mimicking models to improve preclinical screening and reduce this attrition rate. For this purpose, currently, the utilization of organoids is expanding. Tumour organoids can recapitulate tumour microenvironment that is including cancer cells and non‐neoplastic host components. Indeed, tumour organoids, both phenotypically and genetically, resemble the tumour tissue that originated from it. The unique properties of the tumour microenvironment can significantly affect drug response and cancer progression. In this review, we will discuss about various organoid culture strategies for modelling the tumour immune microenvironment, their applications and advantages in cancer research such as testing cancer immunotherapeutics, developing novel approaches for personalized medicine, testing drug toxicity, drug screening, study cancer initiation and progression, and we will also review the limitations of organoid culture systems.

## 1 INTRODUCTION

In cancer research, conventional animal models and cell culture systems have several problems, in the in vitro 2D cell cultures, the cancer‐derived cell lines may acquire considerable genetic mutations and fail to recapitulate the cancer genetic heterogeneity that originated from it.^1^ In addition, the absence of stromal compartments and the lack of normal tissue‐derived cell lines as control are another limitation of 2D cell culture systems.^1^ in vitro 2D cell cultures and in vivo xenografts models are used in the pharmacological intervention, viral transduction and multiplexed drug screening studies. Genetically engineered animal models provide the dynamic context of tumour tissue vasculature and structure.^2^ The generation of genetically engineered animal models is time‐consuming, and it is clear that these animal models do not truly recapitulate pathogenic processes in human.^3^ Both conventional in vivo and in vitro models inefficiently recapitulate the complex immune microenvironment of native tumours.^2^ Humanized immuno‐oncology models are generated by transplanting patient‐derived xenografts (PDXs) into humanized immune system mouse models that bearing human immune cells, but time, cost, throughput and complete immunocompatibility remain challenges.^4^, ^5^ Indeed, patient‐derived tumour xenografts (PDTXs) mimicking the human tumour microenvironment (TME) much better than in vitro 2D culture systems. PDTXs are generated by engraftment of freshly patient‐derived tumour tissue fragments orthotopically or subcutaneously into immunodeficient mice.^1^ Low reproducibility rates of results obtained from animal models, except PDX models that organoids are of human origin, in humans is one of the disappointing problems with cancer therapy development, indeed less than 10% of findings observed in these models can be translated to humans.^6^ Therefore, using human physiological mimicking models are vital to reduce this attrition rate and to improving the preclinical screening.

Organoids are 3D in vitro cultures of tissues with multiple cell lineages, comprising differentiated cells and stem cells, and tissue native construction in vitro.^7^, ^8^ in vitro human organoid culture is a new approach to studying tumour immunobiology and cancer modelling. The large‐scale 3D patient‐derived organoids (PDOs) culture permits the establishment of large tumour biobanks that represent the histological and the genetics of their original malignancies.^9^, ^10^, ^11^ In addition, in forward genetic strategy studies, organoids from induced pluripotent stem cells (iPSCs) or normal tissues can be genetically engineered to gain specific tumour suppressor or oncogene mutations.^12^, ^13^, ^14^, ^15^ Tumour organoids can recapitulate tumour (immune) microenvironment that are including neoplastic cells and non‐neoplastic host components. These properties of the tumour microenvironment play a critical role in tumour behaviour such as carcinogenesis, tumour progression and metastases.^2^ Importantly, these studies have shown that tumour‐derived organoids, both genetically and histologically, be similar to the tumour tissue that originated from it. Currently, a large tumour organoids (3D PDO) biobank and internationally accessible for the research community has been created by cooperation of the Wellcome Sanger Institute and the foundation Hubrecht Organoid Technology, Cancer Research UK, the US National Cancer Institute (NCI) and Human Cancer Models Initiative (HCMI).

In this review, we will discuss about various organoid culture strategies for modelling the tumour immune microenvironment, their applications and advantages in cancer research such as testing cancer immunotherapeutics, developing novel approaches for personalized medicine, testing drug toxicity, drug screening, study cancer initiation and progression, and we will also review the limitations of organoid culture systems.

## 2 ORGANOID CULTURE SYSTEMS FOR MODELLING THE TUMOUR IMMUNE MICROENVIRONMENT

**FIGURE 1:**
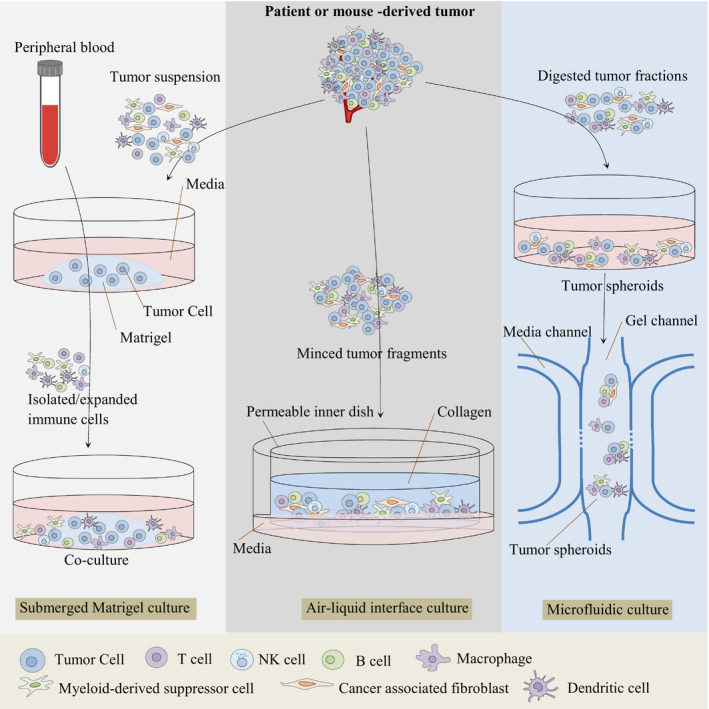
Organoid culture strategies for modeling the tumor (immune) microenvironment

There are various organoid culture strategies for modelling the tumour immune microenvironment including (a) Reconstitution approaches, reconstituted tumour microenvironment immune components, like submerged Matrigel culture, (b) Holistic approaches, native TME immune components, like microfluidic 3D culture, and air‐liquid interface (ALI) culture (Figure 1; Table 1). These methods will be fully explained below. The term TME refers to the complex cellular milieu surrounding cancer epithelium, including mesenchymal‐derived cells such as fibroblasts and pericytes, blood vessels, innate and adaptive immune cellular network and extracellular matrix (ECM). TME immune cells include lymphocytes, myeloid‐derived suppressor cells (MDSCs), macrophages, dendritic cells (DCs), natural killer (NK) cells, mast cells, eosinophils and populate the cancer tissue and can be infiltrated from secondary lymphoid organs, or derived from tissue‐resident cell components. In the tumour tissue, the cellular and humoral components and diverse inflammatory responses of TME support the tumour progression.^16^, ^17^, ^18^ Depending on the organoid culture strategy, TME complex cellular milieu may or may not be preserved in the organoid structure. In approaches where these cells are not preserved, exogenous cellular components can be used. Which are discussed in the following sections. Recapitulation of the vascular system and hypoxia conditions of native TME also are other important aspects of organoid culture.^19^, ^20^ Several studies in recent years addressed the issue of organoid vascularization. Takebe et al^21^ indicated that condensation of mesenchymal cells, endothelial cells, and specific parenchymal cell types leads to the formation of vascularized complex organ buds. After transplantation of these vascularized buds into a mouse, the vasculature was connected to the host circulatory system and the blood was perfused through it. Successful adaptation of this protocol has been reported for generating human complex tissues.^22^ Instead of using a mixture of terminally differentiated cell types, multi‐layered human blood vessels can also be generated via self‐organization from iPS cell‐derived mesodermal progenitor cells (MPCs), which can differentiate into all cell types of blood vessel wall.^20^, ^23^ Wörsdörfer et al^24^ have also described a method to incorporate stromal components in organoids generated from stem cells (that do not have the major components of the organ stroma) by co‐culturing with induced pluripotent stem cell‐derived mesodermal progenitor cells.

**TABLE 1:** Overview of tumour organoid culture systems in cancer research

	Tumour organoid culture approach		
	Submerged Matrigel culture	Microfluidic 3D culture	ALI culture
Samples	Tumour tissues		
Tissue processing before 3D culture	Tissues are dissociated enzymatically and physically	Tissues are dissociated physically and enzymatically; collect 40‐100 μm‐sized spheroid fractions, subsequently maintained in ultra‐low‐attachment plates	Tissues are physically minced into fragments
Culture apparatus	Plate or dish	3D microfluidic culture device	Inner dish, Outer dish, (Transwell plates)
Matrix	Matrigel	Collagen	Collagen
Culture condition	Cell‐Matrigel mixture is plated; medium is added over Matrigel	Spheroid‐collagen mixture is injected into central gel region of device; medium is added into media channels on both sides	Minced tumour tissue fragments are embedded in collagen and plated on bottom collagen layer; medium is added into an outer dish; top of collagen layer is exposed to air
Retained cell types of native tumour tissue in culture	Tumour cells exclusively	Tumour cells, tumour‐infiltrating myeloid and lymphoid cells	Neoplastic cells, native immune cells and stromal fibroblasts
Immune TME	PBMCs, primary leukocytes, TAMs, and DCs can be added in medium	Immune cells can be added in medium; immune TME of primary tissue is faithfully reconstituted	Immune TME of primary tissue is faithfully maintained
Benefits	Easy to enrich and expand	Requires small number of cells and small amount of medium and reagents to test	Preserves diverse immune cells and fibroblasts in TME
Limitations	Lack of non‐neoplastic components	Requires specialized equipment; size limitation; does not reflect recruitment of circulating immune cells into tumour	Creation of uniformly sized organoids; does not reflect recruitment of circulating immune cells into tumour
Refs	43‐46, 50, 52, 54, 77, 79	55, 56, 59	28, 61

### 2.1 Reconstitution approaches: Submerged matrigel culture

In submerged Matrigel culture system, tumour cells obtained from tumour tissue that dissociated enzymatically and physically, culture underneath tissue culture medium in mixed with a flat or dome gel of 3D Matrigel. Depending on the types of cancer tissue, various pathway inhibitors and/or growth factors are added to the culture in this approach.^13^, ^25^, ^26^ Based on tumour type and histology, culture situations can be adapted and customized, but mostly contain some additives, such as R‐spondin (RSPO), WNT3A, epidermal growth factor (EGF) and bone morphogenetic proteins (BMP) inhibitor Noggin, which help the stem cells to maintain their ability of differentiation and self‐renewal.^27^ These additives have also been used for the ALI culture system.^28^ In tumour organoid, the niche factor requirements are mainly determined by genetic mutations and help to local tumorigenicity.^26^ In contrast, during the development of advanced cancer, changing biological behaviours such as the acquisition of TGF‐β/BMP resistance, were observed independently of genetic mutations.^26^ Finally, advanced genetic analysis will detect new genetic mutations accounting for the cancer progression.^26^ In Fujii et al^26^ study, the majority of colorectal cancer (CRC) organoids and all adenoma organoids grew in the absence of exogenous R‐spondin1/Wnt3A. Indeed, R‐spondin1/Wnt3A‐independent organoids carried genetic mutations within the Wnt signalling pathway proteins, including TCF7L2, CTNNB1, and APC.^26^ In the organoid library established by Fujii et al^26^, 22 cancer organoids grew in the absence of exogenous EGF, and in 16 of these EGF‐independent organoids, MAPK signalling pathway mutations were detected. In contrast, no mutation in the RAS/MAPK pathway was observed in the other 6 EGF‐independent colorectal cancer organoids, and 2 of these organoid lines were associated with Epiregulin overexpression.^26^ Fujii et al^26^ showed that 29 CRC organoid lines propagated strongly in the absence of TGF‐β inhibitor. Of these, the known TGF‐β pathway mutations were not detected in 18 CRC organoids.^26^ Similarly, dependency for BMP inhibition was acquired partially through *SMAD4* mutation, but most Noggin‐dependent organoid lines lacked relevant pathway mutations.^26^ Cancer organoids have been grown in rich conditions supplied with niche factors including EGF, WNT, R‐spondin and other factors, whereas, by the alternation of niche factors in the culture medium, functional selection of CRISPR‐induced oncogenic mutations becomes possible,^13^, ^14^ and different cancer subtypes can be grown for establishing cognate PDOs from a mixture of different cancer subtypes.^29^ For instance, *TP53*‐mutant organoids can be selected using a medium containing the MDM2‐P53 complex inhibitor Nutlin‐3, whereas to select *APC*‐mutant organoids, they need to be cultured in a medium without WNT/R‐spondin.^13^, ^14^ In addition, *SMAD4*‐mutant organoids can survive in the absence of TGFβ inhibitor and Noggin but containing TGFβ, and oncogenic PI3KCA^E545K^ and KRAS^G12V/D^ CRISPR knock‐in allows organoid growth without EGF and in presence of EGFR inhibitor gefitinib.^13^, ^14^ Numerous studies have customized different cocktails of growth factors for different organs, including the small and large intestine,^8^, ^27^ breast,^9^ stomach,^30^ salivary gland,^31^ pancreas,^32^ liver,^33^ fallopian tube,^34^ taste buds,^35^ airway,^36^ prostate,^37^ endometrium,^38^ kidney,^39^ ovary,^40^ esophagus ^41^ and skin.^42^

Studies show that PDOs in submerged Matrigel systems can facilitate drug screening and cancer modelling by simulating not only the phenotypic and genetic complexity of cancer tissues, but also by potentially modelling functional individual responses to drug and clinical treatment.^10^, ^11^, ^43^, ^44^, ^45^, ^46^ It should be noted that typical submerged Matrigel PDOs particularly enrich epithelial tumour cells but lose their stromal components and immune cells.^27^ Thus, tumour (immune) microenvironment modelling in this approach requires co‐culture of PDOs with exogenous immune components such as peripheral blood mononuclear cells (PBMCs), primary leukocytes, tumour‐associated macrophages (TAMs), and DCs. Therefore, one of the intrinsic limitations of submerged Matrigel culture is the lack of immune cells, blood vessels and stroma. In many studies, researchers have utilized exogenous stromal cells such as cancer‐associated fibroblasts (CAFs) for the investigation of the tumour microenvironment in this technique.^25^, ^47^, ^48^, ^49^ Co‐culture of human pancreatic ductal adenocarcinoma (PDAC) organoids with CAFs showed that WNT produced by CAF can drive organoid growth in WNT‐nonproducing PDAC subtypes.^25^ Co‐culture of pancreatic stellate cells, a precursor population of CAFs, with PDAC organoids provides evidence for CAF heterogeneity and reveals two distinct CAF subtypes from pancreatic stellate cells: high αSMA‐expressing myofibroblast‐like CAFs that located closely adjacent to tumour cells, and IL‐6 and additional inflammatory mediators secreting CAFs activated by paracrine factors produced from neoplastic cells.^49^ Biffi et al^47^ identified TGFβ and IL‐1 ligands secreted by PDAC organoids co‐cultured with CAFs which promote CAF heterogeneity and induce distinct myofibroblast and inflammatory CAF subtypes, respectively. Understanding the CAF heterogeneity mechanisms is essential for the development of new methods that selectively target tumour‐promoting CAFs.^47^

Reconstitution of organoids has also been performed by various immune cells in submerged Matrigel culture systems. DeNardo et al^50^ by using the mammary epithelial organoids from mouse mammary tumour virus‐polyoma middle T antigen (MMTV‐PyMT) mouse model demonstrated that IL‐4‐expressing CD4^+^ T lymphocytes indirectly induce invasion and subsequent metastasis behaviour of tumour organoid by directly promoting a protumorigenic TAMs phenotype. Co‐culture of primary human pancreatic cancer organoids with peripheral blood lymphocytes and patient‐matched CAFs revealed lymphocyte migrating and infiltration into submerged Matrigel organoids and myofibroblast‐like CAF activation.^48^ A more complex culture system has been designed by co‐culturing of gastric tumour organoids from mouse model with cytotoxic T lymphocytes (CTLs) and bone marrow‐derived DCs pulsed by conditioned media (tumour antigen) collected from cancer organoids, it turned out that in this system, tumour cell apoptosis is increased in gastric tumour organoids by activated CTLs in the presence of PD‐L1 neutralizing antibody.^51^ The mentioned studies suggesting that the reconstitution of tumour organoids with extrinsic immune cells can assist the investigation of immune‐immune and tumour‐immune cell interactions.^51^ Submerged Matrigel models of cancer organoid co‐cultures with DCs in a Helicobacter pylori infection model studies ^52^, ^53^ or autologous peripheral blood lymphocytes to generate tumour‐reactive T cells studies^54^ have been also used.

### 2.2 Holistic approaches: Microfluidic 3D culture

In holistic approaches, the small fragment of tumour tissue is preserved, consisting of stroma endogenous immune cells, tumour epithelium cells, and other cells culture as an intact unit without reconstitution. In a microfluidic culture, organotypic tumour spheroids are cultured as murine‐ or patient‐derived organotypic tumour spheroids (MDOTS/PDOTS) in a mixture of collagen for 5‐9 days in microfluidic devices, spheroids are 40‐100 μm in diameter and typically retain the original tumour tissue cell population and complexity, without reconstitution, including cancer cells and endogenous lymphocyte and myeloid populations.^55^ Microfluidic 3D Culture allows the study of T‐cell infiltration into cancer spheroids by adding T cells into the culture medium, such as Jurkat cells or analysis of endogenous tumour‐immune cell interactions.^56^ Spheroid‐based organotypic cultures in 3D microfluidic devices within collagen gels have been optimized to culture patient‐ or murine‐derived tumours.^57^ Tumour spheroids are added in centre region of the microfluidic device to grow in a mixture of 3D collagen gel and a culture medium also is added to the device from the media channels located on either side of the central region. PDOTS from patient tumour tissues, such as Merkel cell carcinoma and melanoma, and MDOTS from syngeneic immunocompetent murine models can be cultured and evaluated for one to two weeks.^55^, ^58^, ^59^ Studies show that MDOTS/PDOTS retain autologous lymphocytes, myeloid and tumour cells.^55^ Spheroid fractions should be papered in ultra‐low‐attachment tissue culture plates before adding to the Microfluidic device.

It should be noted that the size of the media channels and the size of the central region are different in various types of microfluidic devices that can certainly affect the results of experiments.^59^ The composition of the device is another variable that can reduce the validity of the research results. For example, the AIM Biotech micro‐device, a 3D cell culture chip, is plastic whereas other materials (eg polydimethylsiloxane, PDMS) utilized for ‘home grown’ device construction show different characteristics and properties. It is known the composition has special importance when testing immune checkpoint blockade (ICB) in a mixture with small molecules (generally prepared in dimethyl sulfoxide, DMSO), because PDMS is known for the adsorption of small hydrophobic molecules ^60^ that probably interfere the drug delivery to tumour spheroids and eventually alter the drug efficacy. For the preparation of tumour spheroids (MDOTS/PDOTS),^59^ a cancer tissue specimen is taken and dissociated enzymatically and mechanically. This process ultimately gives a mixture of the macroscopic tumour, spheroids and single cells. This heterogeneous mixture is then passed through 100 μm and 40 μm filters, respectively, to obtain three separate fractions including S1 (>100 μm), S2 (40‐100 μm), and S3 (<40 μm). Then, the S2 fraction is pelleted in ultra‐low‐attachment plates and mixed in collagen to be inoculated into the microfluidic device.

### 2.3 Holistic approaches: Air‐liquid interface culture

In this system, in the first stage, the bottom layer of the collagen gel matrix in the inner dish is prepared.^61^ For the preparation of primary tissues, tissues are removed from other parts and immediately are immersed in ice‐cold medium.^61^ After rinsing the tissue, the tissue is minced into small fragments and then the minced tissue is mixed into collagen solution.^61^ The tissue‐containing collagen gel is poured onto the inner dish with bottom layer gel matrix.^61^ The completed inner dish is placed in a new empty outer dish.^61^ The covered outer dish is transferred to a 37°C incubator and is allowed the gel of the inner dish to solidify.^61^ After solidifying the top layer tissue‐containing gel, media is added to the outer dish that can diffuse into the inner transwell dish through a permeable membrane, and the top layer of tumour fragments‐collagen mixture is exposed directly to air via an ALI, allowing tumour organoids to supply their own oxygen efficiently.^7^, ^15^, ^61^ In this method, in contrast to submerged Matrigel culture, tumour cells are placed alongside endogenous native immune and stromal cells without reconstitution as an intact en bloc.^2^ Initially, ALI organoids culture from various normal tissues, including pancreas, stomach, colon and small intestine, were performed and found that both epithelial and mesenchymal components are incorporate in culture.^7^, ^15^ Subsequently, this approach was established and developed to culture PDOs from patient tumour biopsies, such as renal cell carcinoma (RCC), non‐small cell lung cancer (NSCLC) and melanoma, and mouse cancers in syngeneic immunocompetent mice model.^28^ ALI PDOs maintain not only the genetic mutations and alterations of the original cancer tissue but also the architecture and cellular heterogeneity of the TME. Indeed, both cancer stroma and parenchyma are preserved, including a complex population of endogenous infiltrating immune cells and fibroblasts.^28^

Bourland, et al^62^ (2018) by using the ALI method produced the 3D microvascularized skin as a melanoma model. First, fibroblasts were seeded in tissue culture dishes with peripheral paper anchors in presence of ascorbic acid to stimulate (ECM)‐rich cell sheets formation and facilitate their handling. After that, endothelial cells were seeded on the top of two fibroblast cell sheets, whereas melanoma spheroids and keratinocytes were seeded on a third individual cell sheet. On the 28th day of cell culture, three cell sheets were stacked and placed at the ALI culture system, the melanoma spheroid cell sheet was placed on top of two other cell sheets. On day 38, the model was considered as a mature model based on the histological, stratified epidermis, incorporated tumour cells and well‐formed vasculature networks, and biological properties of model. The mentioned study showed that ALI organoid culture along with reconstitution is a powerful tool to create a human‐mimicking environment for drug screening and other research areas.^62^

In ALI organoid culture, single‐cell sequencing of tumour organoids and original tissue has shown that large regions of cancer tissues are accurately preserved and grown in their native state, and organoids can maintain endogenous immune cell complexity of original tissue including NK cells, B cells, T cells [T cytotoxic, T helper, exhausted (Tex) and regulatory (Treg)] and macrophages.^28^ In addition, in this approach, PDOs can retain the T‐cell receptor (TCR) repertoire of the original fresh cancer tissue, and similar to what has been detected in human cancer tissues, ‘exhausted’ T‐cell phenotypes can be caused by the expansion of T‐cell clonotypes.^28^ ALI PDOs can be cultured from various cancer sites including pancreas, lung, kidney and colon, and these PDOs at least over the short‐term about 30 days, can faithfully recapitulate the histology and mutation complexity of the original cancers.^28^ The immune system components of PDOs are reduced over time and do not persevere beyond ~2 months despite IL‐2 supplementation.^28^ Preservation of the capillary network comprising immune cells could improve modelling of the native biological immune cell circulation, but the passage of fluid‐containing cells through the capillary system would probably remain a challenge.

## 3 APPLICATIONS OF TUMOUR ORGANOIDS

Organoids have many applications in cancer research such as testing cancer immunotherapeutics, developing novel approaches for personalized medicine, testing drug toxicity, study cancer initiation and progression, etc, some of which will be explained in more detail below.

### 3.1 Tumour organoids in Immunotherapies

Predisposition to cancer development during immunodeficiency and inflammatory states illustrates the critical functions of immune systems during oncogenesis.^63^, ^64^ Recently, considerable anticancer efficacies of both pharmacological and cellular immunotherapies on cancer immunomodulation were indisputably confirmed. Immunotherapy agents locally modulate the tumour (immune) microenvironment and/or systemically boost the immune surveillance of the whole body. Immunotherapies include vaccines, pattern recognition receptor (PRR)‐targeted therapies, tumour antigen (TA)‐targeted monoclonal antibodies (mAb) and other non‐specific small molecules.^65^, ^66^ Advance immunotherapy approaches include the following (a) immune checkpoint inhibitors (ICIs),^67^, ^68^, ^69^ monoclonal antibodies against PD‐1/PD‐L1 and CTLA‐4 to unleash CD8+ T‐cell effector functions; and (b) adoptive T‐cell therapies (ACT), chimeric antigen receptor (CAR)‐ and TCR‐T cells, tumour‐infiltrating lymphocyte (TIL) therapy.^70^, ^71^, ^72^ Organoid biobanks have been established from various types of malignancies (Table 2), including ovary,^40^ pancreas,^73^ colon,^11^, ^26^, ^43^ liver,^74^, ^75^ breast,^9^ stomach, rectum ^10^ and prostate,^76^ which are available through institutions such as the HCMI. One of the main limitations of organoids in immunotherapy investigations is that the epithelial‐only patient‐derived organoids are widely available, but their absence of immune cells hinders immunotherapy studies, such as the response to ICIs.

**TABLE 2:** Overview of the currently available human‐patient‐derived tumour organoid (PDO) biobanks

Tumour site	Source	Success rate (%)	N	Refs
Colorectum	Primary tumour	90	20	[11]
		100	55	[26]
	Metastases	70	8	[114]
Rectum	Rectal adenocarcinoma	77	65	[46]
Pancreas	Ductal adenocarcinoma (primary and metastatic specimens)	75	114	[108]
Stomach	Normal, dysplastic, and cancer	>90 normal	63	[10]
	Lymph node metastases	50 cancer		
Liver	Hepatocellular carcinoma	N/A	27	[115]
	Cholangiocarcinoma			
Bladder	Urothelial carcinoma	70	20	[116]
	Squamous‐cell carcinoma			
Prostate	Adenocarcinoma metastases	15‐20	7	[104]
	Circulating tumour cells			
Ovary	Borderline tumours	85	56	[40]
	Clear‐cell carcinoma			
	Endometrioid carcinoma			
	Mucinous carcinoma			
	Serous carcinoma			
Breast	Ductal adenocarcinoma	>80	95	[9]
	Lobular adenocarcinoma			
Esophagus	Oesophageal squamous‐cell carcinoma	71	15	[117]
	Oropharyngeal squamous‐cell carcinoma			
Oral mucosa	Head and neck squamous‐cell carcinoma	65	31	[44]
Endometrium	Normal, endometriosis, hyperplasia, low and high‐grade carcinomas	N/A	72	[118]

### 3.2 Tumour organoids in immune checkpoint inhibitor studies

In 3D microfluidic cultures, the in vivo therapeutic resistance and sensitivity to PD‐1 blockade can be recapitulated by MDOTS/PDOTS for short duration cultures through the evaluation of TIL cytotoxicity against cancer cells by live/dead cell viability assays (eg, PD‐1‐intermediate‐sensitive CT26, PD‐1‐sensitive GL261 and MC38 cancers, Merkel cell carcinoma (MCC), melanoma and PD‐1‐ resistant B16F10).^55^ The assessment of T‐cell cytotoxicity and cytokine profiling against cancer cells in PDOTS‐preserving autologous immune components and cancer cells for a week in a 3D microfluidic device can provide a near‐realistic assessment of patient response to ICI therapy.^55^ On the other hand, ALI organoids co‐retaining various endogenous immune compartments alongside tumour epithelium can establish a suitable model for evaluating responses to ICI treatment by assessing T‐cell functions through fluorescence staining, flow cytometry and tumour‐killing.^77^ In ALI, tumour organoids obtained from mouse tumours transplanted into syngeneic immunocompetent mice (MC38, A20, and B16‐SIY) reveal antigen‐specific clonal CD8‐positive T cells expansion and cytotoxic T cell–mediated tumour cell killing in response to anti‐PD‐1/PD‐L1 antibodies.^78^ Tumour organoid technologies will need prospective validation and correlation with clinical outcome but provide a considerable opportunity for clinical translation through identifying the cohorts significantly responsive to immunotherapies.

### 3.3 Tumour organoids in adoptive cell immunotherapy studies

ACT immunotherapy can be a suitable alternative to ICI therapy. In the ACT, researchers generally utilize bulk autologous TILs or alternatively genetically manipulated T cells such as CAR T‐cell or high‐affinity TCRs recognizing tumour‐specific antigens. In these treatment strategies, antitumour lymphocytes are expanded ex vivo and then the cells are injected into the patient's body.^72^ PDOs, the organoid‐immune cell co‐culture strategies, could be utilized to evaluate CAR T cell–mediated tumour‐specific cytotoxicity in cancer and normal organoids.^79^ However, CD19‐targeted CAR T Cells display striking tumour cell cytotoxicity in haematological malignancies, such as acute lymphoblastic leukaemia ^80^ and B‐cell lymphoma,^81^ but solid tumour cell killing efficiency has not been impressive.^72^ Recently, CRC PDOs have been used to model tumour antigen‐specific cytotoxicity of CAR‐NK92 cells targeting organoids expressing FRIZZLED or EGFRvIII.^79^ However, epithelial‐only submerged Matrigel organoids lack immune cells, but can be exploited as a tumour‐specific antigen source for tumour‐reactive lymphocyte selection. For instance, by co‐cultures of NSCLC or CRC organoids with autologous PBMCs in medium supplemented with anti‐PD‐1, anti‐CD28 and IL‐2 can generate and expand the tumour‐reactive cytotoxic T cells against autologous cancer cells, but not normal cells.^54^ Complex organoid culture systems such as microfluidic or ALI methods that maintaining immune cells could be similarly utilized for translational or mechanistic ACT investigations, although more robust studies are warranted.

In ICI studies, to solve the problems of epithelial‐only submerged Matrigel organoids that is absence of immune cells, immune checkpoint treatment can be performed on epithelial‐only PDOs reconstituted with exogenous immune compartments.^77^, ^82^ It should be noted that expansion of organoids and TILs separately and then co‐culture of epithelial‐only PDOs with autologous TILs, enable TIL migration towards organoids through Matrigel and show tumour cytotoxicity effect, proposing this approach can help to measure the cytotoxic function of TILs.^77^ The possible usage of organoids in immunotherapy screening was confirmed by co‐culture of autologous TILs with organoids exposed with antibodies targeting NKG2A and MICA/B antigens in CRC.^82^ Reconstitution strategies, on the one hand, can improve the reproducibility of experiments by long‐term preservation of the epithelial cells; on the other hand, the co‐culture of single immune cell types with organoids might not completely recapitulate the complex interactions among various immune cell populations following treatment with immunotherapy agents, either alone or in combination.

Recently, it has been shown that peripheral blood can be used as an easily accessible source of tumour‐reactive T cells, an alternative to TILs.^54^ For this purpose, cancer organoids are stimulated with IFNγ to increase antigen presentation and then co‐cultured with autologous T lymphocytes.^54^ Treatment with IFNγ also induce the expression of PD‐L1, a negative regulator of effector T cells, and to eliminate the inhibitory effect of PD‐L1 on the effector T cell, it is necessary to adding blocking antibodies to PD‐1.^54^ To support T‐cell expansion and to provide co‐stimulation, anti‐CD28 and IL‐2 should be added to culture.^54^ It should be noted that the dependency of the induced T‐cell responses on IFNγ should be investigated by the untreated IFNγ control group, as well as the specificity of the response to tumour antigens should be assessed by evaluating the stimulation of T cells with organoids of autologous normal tissue.^54^ It has been reported that T helper cell reactivity is not limited to tumour‐derived organoids but, in some cases, is also stimulated against normal tissue‐derived organoids.^54^ As cross‐reactivity to normal tissue‐derived organoids was observed only for T helper cells and not for cytotoxic T cells, it was suggested that this could be directed against foreign antigens which are present in the culture medium.^54^ Because organoids are grown in murine basement membrane matrix (Geltrex), therefore mouse antigens can be presented to immune cells.^54^ The T helper cell reactivity is only observed in organoids grown in Geltrex or Geltrex‐loaded DCs, but not observed in organoids grown with DC that exposed with healthy or tumour organoids or irradiated cells.^54^ Of note, recently organoids cultures have expanded in synthetic matrices ^83^ and they can be used to escape the stimulatory properties of animal antigens.

In immunotherapy, it is crucial that cancer cells show adequate immunogenicity to provoke an appropriate immune response.^84^, ^85^, ^86^ For cancer cells, the mutational load of a tumour, which represents the amount of neo‐antigens expression, determines the rate of immunogenicity and immune responses.^84^, ^85^, ^87^ In most cases, the potency of immune response triggered by neo‐antigens is insufficient. In vitro immune cells expansion and activation could facilitate the generation of adequate numbers of various immune cells for in vivo use. Recently, for efficiently preserving and expansion of tumour‐specific T cells, thymus organoids are developed that provide a more mimicking physiological environment in vitro.^88^

## 4 TUMOUR ORGANOIDS: STUDY CANCER INITIATION AND PROGRESSION

In tumour cells, to study the origin of mutational signatures remains as a challenge, because numerous different mutational processes are active in cancer development. The genetic stability of normal tissue organoids allows study on the effects of specific mutations in the cancer progression process and mutation signature. Organoids can be utilized to investigate and model tumour initiation and development in specific tissues. Some studies have exploited CRISPR‐Cas9 technology to generate combinations of colorectal cancer driver mutations in normal intestinal tissue organoids to modelling the CRC progression.^13^, ^14^ It was demonstrated that these gene modifications lead to tumour growth that intestinal stem cell niche factors are not involved in it, when inactivating mutations in *TP53*, *APC* and *SMAD4* and activating mutations in *KRAS* are introduced to healthy organoids, tumour growth was independent of the TME factors noggin, R‐spondin‐1, WNT and EGF.^13^, ^14^

It has been displayed that combined inactivating mutations in TP53 and APC are the main drivers of aneuploidy and chromosome instability,^13^ which are hallmarks of colorectal cancer.^89^ Upon subcutaneous xenotransplantation into immunodeficient mice, organoids that had mutations in *APC* (APC knockout, APC^KO^), *P53* (P53^KO^), *KRAS* (KRAS^G12D^) and *SMAD4* (SMAD4^KO^) genes grow as tumours with properties of invasive carcinomas.^13^ Unexpectedly, although driver gene mutations are efficiently sufficient to organoids growth in vivo as invasive cancers, these tumour organoids do not show metastasis, most likely owing to the absence of a native tumour niche. Indeed, when the same CRC organoids were transplanted into the caecal epithelium of mice by an orthotopic approach, spontaneous metastases are seen in the lungs and liver.^90^, ^91^ This orthotopic transplantation approach was also utilized to show that the loss of dependency on specific stem cell niche signals is essential to the ability to metastasize to distant sites,^90^ thus approving former observations in colorectal cancer organoids.^26^ Similar colorectal cancer progression models were generated using RNAi‐based technologies in ALI colonic and mouse small intestinal organoids.^15^

The current revolution in genome‐editing strategies such as RNAi and CRISPR‐Cas9 has made it possible to ‘repairing’ the genetic causes of certain diseases. Organoid technology could likely be facilitated to survey whether the repair of a specific tumour‐causing mutation returns the tumorigenic phenotype. Although cancer is a more complex genetic disease with hundreds of gene mutations, it was recently demonstrated that repair the APC mutation restores intestinal crypt homeostasis in a mouse colorectal cancer model and in organoids derived from this model.^92^, ^93^ It would be interesting to investigate whether repairing the several driver gene mutations at the same time in tumour organoids lead to a huge tumour regression.

## 5 TUMOUR ORGANOIDS IN DRUG TOXICITY STUDIES

The ability of organoids generation from both tumour and healthy tissues is one of the main advantages of using organoid culture in drug development studies, which provides a powerful tool for selecting drugs that target cancer cells specifically but do not damage the healthy cells. As a result, toxicity in patients is likely reduced. Drug‐induced hepatotoxicity is the main reason for the failure of the translation of the drugs to clinical trials.^94^ So recently, liver organoids culture has developed that could facilitate the preclinical screening of the hepatotoxicity of drugs.^95^, ^96^ The major mechanism of drug‐induced liver injury is mediated through cytochromes P450 (CYPs), and it is suggesting that expression of these enzymes in liver organoids be close to physiological levels upon induced differentiation.^33^, ^96^ Similarly, iPSC‐derived cardiac organoids could be exploited for cardiac toxicity testing,^97^, ^98^ and iPSC‐derived kidney organoids were recently utilized for nephrotoxicity screening.^99^ Researchers also can investigate the exciting possibility of assessing the potential cytotoxicity of healthy donor‐derived T cells on patient‐derived tumour organoids after the selection of neo‐antigen‐specific T cells derived from healthy blood donors.^100^

## 6 ORGANOIDS IN PERSONALIZED CANCER TREATMENT AND DRUG DEVELOPMENT

Although high‐throughput 2D cell line screening platforms have provided major insights into the genetic background of experimental components response,^101^ but their poorly recapitulation of the original tumour tissue may be the main cause of the high failure rate of novel anticancer drugs in clinical trials.^102^, ^103^ Tumour organoids can better recapitulate original tumour tissue heterogeneities and might be superior models to recognize and screen newly discovered drugs. High‐throughput patient‐derived organoids drug screening technology is in its early stages. Small‐scale drug testing on organoid bio‐banks conducted so far have had satisfactory results.^9^, ^11^, ^104^ Profiling of patient‐derived organoids may disclose fundamental genetic and/or epigenetic variations that cause drug resistance, which can be utilized to classify patients into specific treatment regimens. By genetic characterization of organoid cultures in drug screening studies, the genetic basis of tumour response to a drug can be determined. Patient and healthy donor‐derived organoids can be stored in living organoid biobanks after cryopreservation. The generation of organoids from tumour and healthy tissues from the same patient provides the chance to develop drugs with low toxicity by selecting drugs that specifically kill cancer organoid cells whereas leaving healthy organoids unharmed. Recently, the organ‐specific mutation spectrum can be studied by whole‐genome sequencing of the clonal organoids culture from various healthy tissues.^105^ Organoid culture can be also utilized to investigate the intratumour heterogeneity by culturing clonal organoids from distinct regions of the same tumour. Thus, region‐specific mutation spectra can be shown by whole‐genome sequencing of the clonal organoid cultures.^106^ Using these approaches, organoids can be used to survey the effects of the mutation profiles on drug response. Another surprising application of organoids came from the treatment of the same tumour clones with different anticancer drugs separately for selecting the most effective drug in clinical practice uses.^107^ Conventional Matrigel PDOs can be exploited as a hopeful platform for assessing the functional responses of patients with cancer to anticancer drugs ^45^, ^46^, ^108^ and combined chemoradiotherapy.^45^, ^46^ However, to investigate its full effectiveness, more research is needed to include survey on the effects of tumour heterogeneity, observe reproducibility in validation cohorts, study on rapid real‐time analysis and, eventually, observe overall survival. Nevertheless, the application of tumour organoids in predicting personalized responses to conventional cancer therapy agents is an active area of investigation.

## 7 TUMOUR ORGANOIDS

### 7.1 Pure tumour material

Tumour tissue‐derived organoids do not usually grow faster than their matching healthy organoid tissues, and, unexpectedly, in many cancers the organoid growth rate is even slower, probably due to higher rates of the mitotic failure process and following cell death.^13^, ^109^ Therefore, the overgrowth of tumour organoids can be occurred by remaining healthy tissue fragments in tumour biopsy samples, which should be avoided. So, it is necessary that tumour organoids are cultured using either pure cancer tissue materials or grow the tumour specimens under selective culture conditions. For instance, in the vast majority of CRCs, the Wnt signalling pathway proteins demonstrate gain‐of‐function mutations.^110^ In this type of cancer, pure tumour organoid material for culture can be achieved by using WNT and R‐spondins free culture media,^41^ which these factors are needed for the growth of healthy tissue‐derived organoids. Similarly, cancers with activating mutations in the EGF receptor signalling pathway can be selected by EGF‐free culture medium.^13^, ^26^, ^111^ Nutlin‐3, which inhibits the interaction between p53 and its negative regulator MDM2 by blocking the p53‐binding domain of MDM2, has been exploited to remove healthy fragments from *TP53*‐mutant tumour organoids.^9^, ^13^ When such selection approaches are not accessible, using pure tumour materials is a prerequisite.

## 8 LIMITATIONS AND PERSPECTIVES

Although organoid technology is promising at first glance, but organoids have their limitations as well. For instance, compared with 2D culture systems, organoid‐based approaches require huge time, materials and reagent. The lack of immune cells, stroma and blood vessels is also one of the intrinsic restrictions of organoid systems.^29^ The requirement for mouse‐derived ECM and foetal calf serum (which is essential for the production of WNT conditioned medium in some organoids ^112^, ^113^) is required for the organoid culture which as undefined external factors can influence the test results.^25^ Another imaginable limitation may be that advanced cancers derived organoids often grow more slowly than healthy tissue‐derived organoids, which probably leads to the overgrowth of tumour organoids contaminated with healthy tissue materials.^13^, ^109^ This low growth rate of tumour organoids could be due to a much higher rate of mitotic failure and subsequent cell death.

Despite these restrictions, organoid cultures can be efficiently generated from individual‐patient‐derived tumour tissue, making them as a more physiologically mimicking model for translational applications and the development of personalized cancer medicine. It will be crucial that organoids can be generated and expanded efficiently to allow drug testing in a clinically meaningful time window. Although the use of organoid culture in the diagnosis of cancer relatively has been shown, the predictive value of tumour organoids in drug responses will have to come from ongoing trials. The finding of a study comparing drug responses of the patients in the clinic with the responses of gastrointestinal tumour‐derived organoids are very promising.^43^ Optimizing drug testing strategies in terms of robustness and sensitivity will be crucial before organoid‐based precision medicine can be implemented in the clinic.

## CONFLICT OF INTEREST

The authors confirm that there are no conflicts of interest.

## AUTHOR CONTRIBUTION

**Tao Xia:** Funding acquisition (lead); Visualization (supporting); Writing‐original draft (equal). **Wen‐Lin Du:** Visualization (lead); Writing‐original draft (equal); Writing‐review & editing (supporting). **Xiao‐Yi Chen:** Conceptualization (equal); Project administration (equal); Writing‐review & editing (equal). **You‐Ni Zhang:** Conceptualization (equal); Project administration (equal); Writing‐review & editing (equal).

## Data Availability

Data sharing not applicable to this article as no datasets were generated or analysed during the current study.
